# Diagnosis and Management of Oropharyngeal Dysphagia and Its Nutritional and Respiratory Complications in the Elderly

**DOI:** 10.1155/2011/818979

**Published:** 2010-08-03

**Authors:** Laia Rofes, Viridiana Arreola, Jordi Almirall, Mateu Cabré, Lluís Campins, Pilar García-Peris, Renée Speyer, Pere Clavé

**Affiliations:** ^1^Centro de Investigación Biomédica en Red de Enfermedades Hepáticas y Digestivas (Ciberehd), Instituto de Salud Carlos III, 08036 Barcelona, Spain; ^2^Unidad de Exploraciones Funcionales Digestivas, Hospital de Mataró, 08304 Mataró, Spain; ^3^Unidad de Cuidados Intensivos, Hospital de Mataró, 08304 Mataró, Spain; ^4^Centro de Investigación Biomédica en Red de Enfermedades Respiratorias (Ciberes), Instituto de Salud Carlos III, 07110 Mallorca, Spain; ^5^Unidad Geriátrica de Adultos, Hospital de Mataró, 08304 Mataró, Spain; ^6^Servicio de Farmacia, Hospital de Mataró, 08304 Mataró, Spain; ^7^Nutrition Unit, Hospital General Universitario Gregorio Marañón, 28007 Madrid, Spain; ^8^Department of Otorhinolaryngology and Head and Neck Surgery, Maastricht University Medical Centre, 6200 MD Maastricht, The Netherlands; ^9^Comprehensive Cancer Centre West, 2316 XB Leiden, The Netherlands

## Abstract

Oropharyngeal dysphagia is a major complaint among older people. Dysphagia may cause two types of complications in these patients: (a) a decrease in the efficacy of deglutition leading to malnutrition and dehydration, (b) a decrease in deglutition safety, leading to tracheobronchial aspiration which results in aspiration pneumonia and can lead to death. Clinical screening methods should be used to identify older people with oropharyngeal dysphagia and to identify those patients who are at risk of aspiration. Videofluoroscopy (VFS) is the gold standard to study the oral and pharyngeal mechanisms of dysphagia in older patients. Up to 30% of older patients with dysphagia present aspiration—half of them without cough, and 45%, oropharyngeal residue; and 55% older patients with dysphagia are at risk of malnutrition. Treatment with dietetic changes in bolus volume and viscosity, as well as rehabilitation procedures can improve deglutition and prevent nutritional and respiratory complications in older patients. Diagnosis and management of oropharyngeal dysphagia need a multidisciplinary approach.

## 1. Definition and Prevalence

Dysphagia is a symptom that refers to difficulty or discomfort during the progression of the alimentary bolus from the mouth to the stomach. From an anatomical standpoint dysphagia may result from oropharyngeal or esophageal dysfunction and from a pathophysiological standpoint from structure-related or functional causes [1, 2]. The prevalence of oropharyngeal functional dysphagia is very high: it affects more than 30% of patients who have had a cerebrovascular accident; 52%–82% of patients with Parkinson's disease; 84% of patients with Alzheimer's disease, up to 40% adults aged 65 years and older, and more than 60% of elderly institutionalized patients [2, 3]. Increase in the percentage of older persons is one of the principal demographic characteristics of the population of developed countries. In Europe, more than 17% of the citizens are older than 65 years. In the last decade, this group has increased by 28% whereas the rest of the population has only grown 0.8 % [1]. It has been estimated that 16,500,000 US senior citizens will require care for dysphagia by the year 2010 [4]. In spite of its enormous impact on the functional capacity, health, and quality of life of the older persons who suffer it, oropharyngeal dysphagia is underestimated and underdiagnosed as a cause of symptoms and major nutritional and respiratory complication in older patients. Oropharyngeal dysphagia fulfills most criteria to be recognized as a major geriatric syndrome as its prevalence is very high in geriatric patients and results in multiple diseases, risk factors, and precipitating diseases [5]. The current state of the art with oropharyngeal dysphagia management in older patients aims at identifying patients at risk for dysphagia early, by assessing alterations in the biomechanical events of oropharyngeal swallow response, attempting to prevent and treat the potential complications of dysphagia such as aspiration pneumonia (AP) and malnutrition, and recognizing oropharyngeal dysphagia as a major geriatric syndrome.

Identification of functional oropharyngeal dysphagia as a major neurological and geriatric syndrome will cause many changes in the provision of medical and social services in the near future. Education of health professionals on diagnosis and treatment of dysphagia and its complications, early diagnosis, development of specific complementary explorations in the clinical setting, improvement in therapeutic strategies to avoid aspirations and malnutrition, and research into its pathophysiology are the cornerstones to allow maximal recovery potential for older patients with functional oropharyngeal dysphagia.

## 2. Pathophysiology

**Figure 1:**
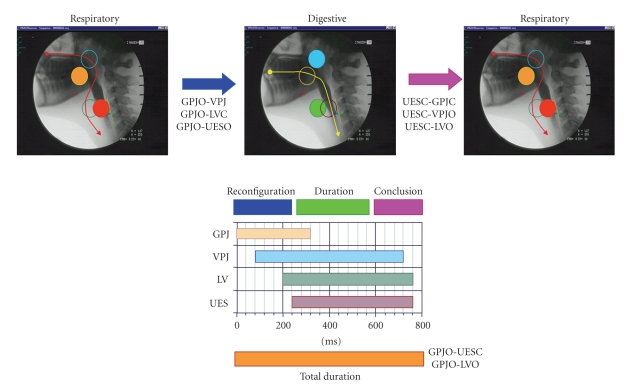
Configuration of the oropharynx during swallow response. Each phase of the response (reconfiguration, duration and conclusion) is defined by opening (O) or closing (C) events occurring at the glossopalatal junction (GPJ), velopharyngeal junction (VPJ), laryngeal vestibule (LV), and upper esophageal sphincter (UES).

Oropharyngeal dysphagia may result from a wide range of *structural alterations *that may impair bolus progression. Most common structural abnormalities include esophageal and ENT tumors, neck osteophytes, postsurgical esophageal stenosis, and Zenker's diverticulum [2]. Dysphagia may also be a side effect in patients with head & neck cancer undergoing radiotherapy. However, oropharyngeal dysphagia in the elderly is more frequently a *functional disorder of deglutition* affecting oropharyngeal swallow response caused by aging, stroke, or associated with systemic or neurological diseases. In biomechanical terms, the oropharyngeal swallow response (OSR) consists of the temporal arrangement of oropharyngeal structures from a respiratory to a digestive pathway, the transfer of the bolus from the mouth to the esophagus, and the recuperation of the respiratory configuration [6, 7] (Figure 1). Sensory input by physicochemical properties of the bolus is required during bolus preparation and trigger and modulate the swallow response. Taste, pressure, temperature, nocioceptive, and general somatic stimuli from the oropharynx and larynx are transported through cranial nerves V, VII, IX and X to the central pattern generator (CPG), within the nucleus tractus solitarius (NTS), where they are integrated and organized with information from the cortex. Swallowing has a multiregional and assymmetrical cerebral representation in caudal sensorimotor and lateral premotor cortex, insula, temporopolar cortex, amygdala, and cerebellum. This observation explains why 30%-50% of unilateral hemispheric stroke patients will develop dysphagia [8]. Once activated, the CPG triggers a swallow motor response involving motor neurons in the brainstem and axons traveling through the cervical spinal cord (C_1_-C_2_) and cranial nerves (V, VII, IX, to XII) [7]. 

**Figure 2:**
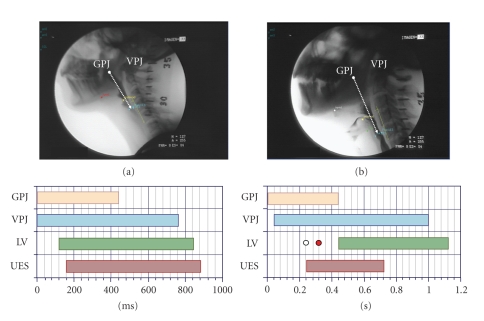
Videofluoroscopic pictures and oropharyngeal swallow response during the ingestion of a 5 mL nectar bolus in: (a) a healthy individual; (b) an older patient with neurogenic dysphagia and aspiration associated with stroke. An increased total duration of the swallow response may be seen, as well as a delayed closure of the laryngeal vestibule and delayed aperture of the upper sphincter. The white dot indicates the time when contrast penetrates into the laryngeal vestibule, and the red dot indicates passage into the tracheobronchial tree (aspiration). GPJ = glossopalatal junction, VPJ = velopalatal junction, LV = laryngeal vestibule, UES = upper esophageal sphincter.

Duration of the swallow response in healthy humans is in the range of 0.6–1 s [7]. Healthy subjects presented a short reaction time in the submental muscles [9], short swallow response (GPJO-LVO < 740 ms), fast laryngeal vestibule closure (LVC < 160 ms), and fast upper esophageal sphincter opening (UESO < 220 ms) [10]. In contrast, the swallow response is impaired in older people, especially in patients with neurogenic dysphagia [9–11]. Older patients have prolonged reaction time in the submental muscles [9], and overall duration of OSR in these subjects is significantly longer than in healthy volunteers due to delay in the early phase of oropharyngeal reconfiguration from a respiratory to a digestive pathway [10]. We found prolonged intervals to LVC and UESO were the key abnormalities of swallow response, doubling that of healthy subjects and leading to unsafe deglutition and aspiration in neurological older patients (Figure 2) [10, 11]. This delayed swallow response in the elderly can be attributed to an impairment of sensations [12, 13], a decrease in the number of neurons in the brain, and a delay in the synapse conduction in the afferent inputs to the central nervous system (SNC) caused by aging [9] and by other risk factors for dysphagia like neurodegenerative diseases or stroke [1, 14]. Other conditions such as delirium, confusion and dementia, and the effects of sedative, neuroleptic, or antidepressant drugs, can also contribute to impaired swallow response in frail older patients [14]. Transfer of the bolus from the mouth through the pharynx is mainly caused by the squeezing action of the tongue [15]. Older adults present lingual weakness, a finding that has been related to sarcopenia of the head and neck musculature and frailty [16]. Tongue propulsion is assessed by direct measurements with oral sensors [16] or by videofluoroscopic studies which measure the bolus velocity and kinetic energy during swallow [10]. Older adults generate lower maximum isometric pressures than younger adults [16]. We found young healthy adults present high bolus velocity (>35 cm/s) and strong bolus propulsion forces (>0.33 mJ) [10]. In contrast, older people with oropharyngeal dysphagia present impaired tongue propulsion forces (<0.14 mJ) and slower bolus velocity (<10 cms/s) [10]. Therefore, dysphagia in the elderly is associated with impairment in efficacy and safety of swallow caused by weak tongue propulsion and prolonged and delayed swallow response. Pathogenesis of impaired safety is related to a delay in several physiologic protective reflexes in oropharyngeal reconfiguration (mainly laryngeal vestibule closure) caused by a slow neural swallow response and is associated with several risk factors such as aging, neurodegenerative diseases, confusion, dementia, and drugs (Figure 7). Pathogenesis of impaired efficacy is related to alterations in bolus propulsion caused by a weak muscular tongue squeeze associated to sarcopenia and weakness [1].

## 3. Diagnosis

In many hospitals there is a big discrepancy between the high prevalence, morbidity, mortality, and costs caused by nutritional and respiratory complications of functional oropharyngeal dysphagia and the restricted availability of human and material resources dedicated to dysphagic patients. Dysphagia with oropharyngeal aspiration is not usually considered an etiologic factor in older patients with community-acquired pneumonia [17, 18] or with malnutrition [19]. Diagnosis and management of oropharyngeal dysphagia needs a **multidisciplinary approach**. A *dysphagia multidisciplinary team *should include several professional domains: nurses, speech-swallow therapists, gastroenterologists, ENT specialists, neurologists, surgeons, rehabilitation physicians, dietitians, radiologists, and geriatricians. The goals of a multidisciplinary dysphagia team include: (a) early identification of older patients with dysphagia; (b) diagnosis of any medical or surgical etiology for dysphagia that may respond to specific treatment; (c) characterization of specific biomechanical events responsible for functional dysphagia in each patient; and (d) the design of a set of therapeutic strategies to provide patients with safe and effective deglutition, or the provision of an alternative route to oral feeding based on objective and reproducible data [2, 19]. The involvement of patient's family in the diagnostic and therapeutic process is of capital importance. Once a diagnosis of functional oropharyngeal dysphagia has been established, the goal of the diagnostic program is to evaluate two deglutition-defining characteristics: (a) *efficacy, *the patient's ability to ingest all the calories and water he or she needs to remain adequately nourished and hydrated; and (b) *safety, *the patient's ability to ingest all needed calories and water with no respiratory complications [1, 2, 10, 19]. To assess both characteristics of deglutition two groups of diagnostic methods are available (a) *clinical methods *such as deglutition-specific medical history and clinical examination, usually used as screening methods; and (b) the exploration of deglutition using *specific complementary studies *such as videofluoroscopy. 

**Figure 3:**
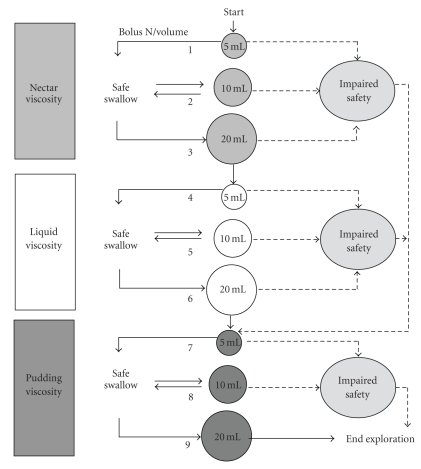
Algorithms of bolus volume and viscosity administration during V-VST. The strategy of the V-VST aims at protecting patients from aspiration by starting with nectar viscosity and volumes were increased from 5 mL, to 10 mL and 20 mL boluses in a progression of increasing difficulty. When patients completed the nectar series without major symptoms of aspiration (cough and/or fall in oxygen saturation ≥3%), a less “safe” liquid viscosity series was assessed also with boluses of increasing difficulty (5 mL to 20 mL). Finally, a more “safe” pudding viscosity series (5 mL to 20 mL) was assessed using similar rules. If the patient presents a sign of impaired safety at nectar viscosity, the series is interrupted, the liquid series is omitted, and a more safe pudding viscosity series is assessed. If the patient presents a sign of impaired safety at liquid viscosity, the liquid series is interrupted and the pudding series is assessed (Figure 1(C)).

**Figure 4:**
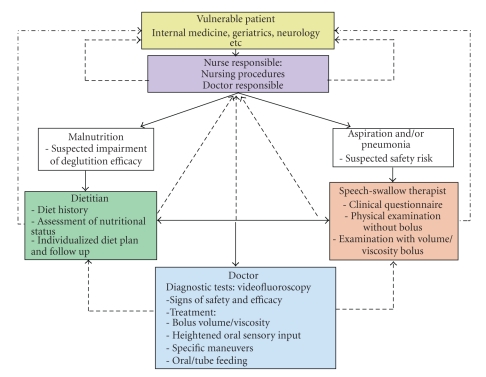
Algorithm for screening, diagnosis and treatment of oropharyngeal functional dysphagia at the Hospital de Mataró. Barcelona. Spain. Note the involvement of several professional domains of the dysphagia multidisciplinary team and the vertical and horizontal flows of information. The continuous black lines indicate the diagnostic screening strategy of patients at risk; the broken lines indicate flow of information on patient status, and broken dotted lines indicate therapeutic interventions.

*Clinical screening* for oropharyngeal dysphagia should be low risk, quick, and low cost and aim at selecting the highest risk patients who require further assessment. Current methods for clinical screening of dysphagia are, for example, the water swallow test [20], the 3-oz water test developed in the Burke Rehabilitation Center [21], the timed swallow test [22], and the standardized bedside swallow assessment (SBSA) [23, 24]. Patients are asked to drink 50 mL [25], 3 oz [21], 150 mL [22], or 60 mL [23, 24] water from a glass without interruption. Coughing during or after completion or the presence of a postswallow wet-hoarse voice quality, or swallow speed of less than 10 mL/ are scored as abnormal. These clinical bedside methods can detect dysphagia, although with differing diagnostic accuracy. The Burke's 3-oz water swallow test identified 80% of patients aspirating during subsequent VFS examination (sensitivity 76%, specificity 59%) [21]. The SBSA showed a variable sensitivity (47% to 68%) and specificity (67% to 86%) in detecting aspiration when used by speech swallow therapists or doctors [23, 24]. Note that these screening procedures involve continuous swallowing of quite large amounts of liquid and may place the patient at high risk for aspiration. Furthermore, many of these studies on bedside screening lack methodological quality and, therefore, the psychometric properties of the screening procedure being studied cannot be determined accurately [26]. Our team developed a safer clinical method (the volume-viscosity swallow test, V-VST) using a series of 5–20 mL nectar, liquid and pudding boluses sequentially administered in a progression of increasing difficulty (Figure 3). Cough, fall in oxygen saturation ≥3%, and changes in quality of voice were considered clinical signs of impaired safety, and piecemeal deglutition and oropharyngeal residue, signs of impaired efficacy. The V-VST is a safe, quick, and accurate clinical method with 88.2% sensitivity for impaired safety, 100% sensitivity for aspiration and up to 88.4% sensitivity for impaired efficacy of swallow [2]. Figure 4 shows the algorithm for management (screening, diagnosis, and treatment) of oropharyngeal dysphagia at the Hospital de Mataró, Barcelona, Spain [19]. The V-VST is considered to be a highly adequate instrument for screening for dysphagia and agrees with the recommendations stated in the systematic review on bedside screening for dysphagia by Bours et al. [26] to combine a water test and pulse oximetry using coughing, choking, and voice alteration as endpoints. The use of different viscosities in the V-VST can be considered to be an improvement compared to a simple water test using only liquid.

Videofluoroscopy(VES) is the gold standard to study the oral and pharyngeal mechanisms of dysphagia [2, 27]. If no VFS is available, fiberoptic endoscopic evaluation of swallowing (FEES) may be used as a valuable screening instrument instead [28]. VFS is a dynamic exploration that evaluates the safety and efficacy of deglutition, characterizes the alterations of deglutition in terms of videofluoroscopic symptoms, and helps to select and assess specific therapeutic strategies. Technical requirements for clinical VFS are an X-ray tube with fluoroscopy and a videotape recorder; and there are computed-assisted methods of analysis of images allowing quantitative temporal and spatial measurements [10]. Main observations during VFS are done in the lateral plane while swallowing 3–20 mL boluses of at least three consistencies: liquid, nectar, and pudding. We keep the patient at a minimal risk for aspiration by starting the study with low volumes and thick consistencies, introducing liquids and high volumes as tolerated [10]. Major signs of impaired efficacy during the oral stage include apraxia and decreased control and bolus propulsion by the tongue. Many older patients present deglutitional apraxia (difficulty, delay, or inability to initiate the oral stage) following a stroke. This symptom is also seen in patients with Alzheimer's, dementia and patients with diminished oral sensitivity. Impaired lingual control (inability to form the bolus) or propulsion results in oral or vallecular residue when alterations occur at the base of the tongue. The main sign regarding safety during the oral stage is glossopalatal (tongue-soft palate) seal insufficiency, a serious dysfunction that results in the bolus falling into the hypopharynx before the triggering of the oropharyngeal swallow response and while the airway is still open, which causes predeglutitive aspiration [2, 29]. Videofluoroscopic signs of safety during the pharyngeal stage include penetrations and/or aspirations. Penetration refers to the entering of contrast into the laryngeal vestibule within the boundaries of the vocal cords. When aspiration occurs, contrast goes beyond the cords into the tracheobronchial tree (Figure 2(b)). The potential of videofluoroscopy regarding image digitalization and quantitative analysis currently allows accurate swallow response measurements in patients with dysphagia (Figure 2). A slow closure of the laryngeal vestibule and a slow aperture of the upper esophageal sphincter (as seen in Figure 2(b)) are the most characteristic aspiration-related parameters [10, 11]. Penetration and aspiration may also result from an insufficient or delayed hyoid and laryngeal elevation, which fail to protect the airway. A high, permanent postswallow residue may lead to postswallow aspiration, since the hypopharynx is full of contrast when the patient inhales after swallowing, and then contrast passes directly into the airway [2, 29]. Thereafter, VFS can determine whether aspiration is associated with impaired glossopalatal seal (predeglutitive aspiration), a delay in triggering the pharyngeal swallow or impaired deglutitive airway protection (laryngeal elevation, epiglottic descent, and closure of vocal folds during swallow response), or an ineffective pharyngeal clearance (postswallowing aspiration) [2].

## 4. Complications of Oropharyngeal Dysphagia

**Figure 5:**
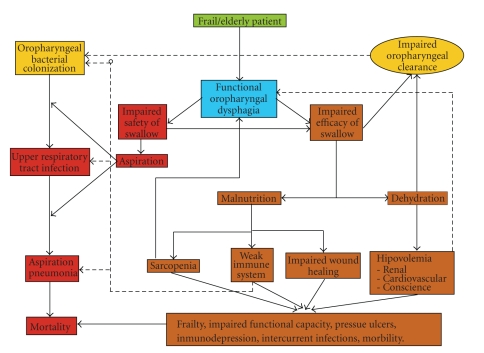
Pathophysiology of nutritional and respiratory complications associated to oropharyngeal dysphagia in elderly patients.

The severity of oropharyngeal dysphagia varies from moderate difficulty to complete inability to swallow. Oropharyngeal dysphagia may give rise to two groups of clinically relevant complications in older people: (a) malnutrition and/or dehydration caused by a decrease in the efficacy of deglutition, present in up to 25%–75% patients with dysphagia; (b) choking and tracheobronchial aspiration caused by the decrease in deglutition safety and which results in pneumonia in 50% of cases, with an associated mortality of up to 50% [1, 2]. A recent 10-year review found a 93.5% increase in the number of hospitalized older patients diagnosed with AP, while other types of pneumonia in the elderly decreased [30]. Figure 5 summarizes the pathophysiology of complications related to dysphagia in the elderly.

### 4.1. Malnutrition and Dehydration

Impairment in swallowing efficacy may reduce oral feeding and lead to malnutrition unless nutritional status is monitored and specific dietetic strategies are introduced to enhance caloric intake. Up to 30% of our neurological patients and up to 55% of our frail older patients with dysphagia present or are at risk of malnutrition with a strong relationship between severity of dysphagia and incidence of malnutrition [1, 10]. A recent resolution of the Council of Europe on food and nutritional care in hospitals claimed that undernutrition among hospital patients leads to extended hospital stays, prolonged rehabilitation, diminished Quality of Life, and unnecessary health care costs; and identified functional oropharyngeal dysphagia as a major contributor to malnutrition [31]. Recommendations from this resolution affecting dysphagia included (a) the development of dietary management at national levels as well as national descriptors for texture modification, (b) documentation and assessment of food intake, (c) detailed food service contracts to include texture-modified menus, (d) meal serving system adjusted to patients, and (e) informing and involving patients/families in the process by giving them help and guidance in ordering and consuming food. Recent guidelines on the indications of enteral nutrition in geriatrics also highlighted the role of dysphagia causing undernutrition in older patients [31]. Dehydration is also a frequent complication of dysphagia in elderly patients with oropharyngeal dysphagia [32, 33]. Dehydration and increased plasma osmolarity showed a significant association with mortality in older stroke patients [33]. Figure 5 shows the pathophysiology of complications of dysphagia associated with malnutrition and dehydration. We previously found that malnutrition in patients with neurogenic dysphagia was uniformly marasmic [10]. We believe all older patients with oropharyngeal dysphagia need nutritional assessment to detect those with malnutrition or at nutritional risk. There are several nutritional screening tools developed for assessing different populations. Mininutritional Assessment (MNA) [34] is a reliable tool for evaluating the nutritional status of older people. It is composed of 18 items covering anthropometric assessment (weight, height, and weight loss), general assessment (lifestyle, medication and mobility), dietary assessment (number of meals, food, and fluid intake), and autonomy of eating and is self assessed (self-perception of health and nutrition). In a very recent study using the MNA in older patients with dysphagia and pneumonia we found the prevalence of malnourished patients (36.8%) and patients at risk of malnutrinion (55.3%) was significantly higher than in older patients without dysphagia [35]. If a patient is at nutritional risk or malnourished, nutritional counselling will be given to improve the oral feeding. This is the first nutritional intervention previous to nutritional support. In some circumstances, nutritional counselling is not enough to maintain or recover proper nutritional status and oral nutritional supplements (ONSs) are indicated. Milne [36] reviewed 55 randomized control trials that studied the clinical and nutritional benefits of ONS in older patients on hospital admission, at home, and in nursing homes. The authors concluded that ONS can improve nutritional status and reduce morbimortality in malnourished patients during hospital admission. The scientific evidence does not support ordinary supplementation in older people at home or older well-nourished patients in any situation (hospital, home, or nursing home). However, in patients with stroke and dysphagia, the FOOD study [37] evaluated the effect of systematically adding an oral supplement to the hospital diet. These data did not support indiscriminate use of ONS in patients with stroke and it must be prescribed only in malnourished patients on admission or those in whom nutritional status was impaired.

### 4.2. Respiratory Complications: Aspiration Pneumonia

The incidence and the prevalence of AP in the community are poorly defined. They increase in direct relation with age and underlying diseases. The risk of AP is higher in older patients because of the high incidence of dysphagia [38]. In elderly nursing home residents with oropharyngeal dysphagia, AP occurs in 43%–50% during the first year, with a mortality of up to 45% [27]. We recently studied 134 older patients (>70 yr) consecutively admitted with pneumonia in an acute geriatric unit in a general hospital. Of the 134 patients, 53% were over 84 years old and 55% presented clinical signs of oropharyngeal dysphagia; the mean Barthel score was 61 points, indicating a frail population. Patients with dysphagia were older, showed lower functional status, higher prevalence of malnutrition and comorbidities and higher Fine's pneumonia severity scores. Patients with dysphagia had higher mortality at 30 days (22.9% versus 8.3%, *P* = .033) and at 1 year of follow-up (55.4% versus 26.7%, *P* = .001). Therefore, oropharyngeal dysphagia is a highly prevalent clinical finding and an indicator of disease severity in older patients with pneumonia [35]. 

The pathogenesis of aspiration pneumonia has been recently revised [17, 18] and presumes the contribution of risk factors that alter swallowing function, cause aspiration and predispose the oropharynx to bacterial colonization. Aspiration observed at VFS is associated with a 5.6–7-fold increase in risk of pneumonia [39]. Up to 45% of older patients with dysphagia presented penetration into the laryngeal vestibule and 30%, aspiration, half of them without cough (silent aspiration); and 45%, oropharyngeal residue [1]. It is accepted that detection of aspiration by VFS is a predictor of pneumonia risk and/or probability of rehospitalization [27]. It is also well known that not all patients who aspirated during VFS develop pneumonia. Impairment in host defenses such as abnormal cough reflex [17, 40], impaired pharyngeal clearance [25], amount and bacterial concentration of aspirate, and weakened immune system also strongly contributed to the development of AP [18]. Impairment of cough reflex increases the risk of AP in stroke patients [40]. Several risks factors contribute to oropharyngeal colonization such as the following (1) Older age, as swallow response, cough reflex, and breathing coordination are impaired in older people. (2) Malnutrition, poor nutritional status is a marker of a population highly susceptible to acquire pneumonia in the elderly as malnutrition depresses the immune system. (3) Smoking status, number of cigarettes smoked per day, and lifetime smoking, and (4) Poor oral hygiene. Probably the most common infectious sequelae of poor oral health in seniors, particularly those who reside in nursing homes, is AP. The oral environment in people who still have teeth is quite different from the flora that thrive in the toothless person but all of them result in oropharyngeal colonization by potential respiratory tract pathogens. (5) Antibiotics, it has been suggested that inappropriate antibiotic treatment could be a risk factor for pneumonia. In some patients who are smokers or with chronic bronchitis, the use of antibiotics in the previous 3 months may provoke a variety of respiratory flora, predisposing to opportunistic infection with colonization of more aggressive organisms, which could be causative pathogens of AP. (6) Dry mouth, many medications reduce salivary flow or create xerostomia as a side effect. This creates a favourable environment for growth of bacteria that are pathogenic to the lungs if aspirated. (7) Immunity, older adults can have reduced oropharyngeal clearance, reduced numbers of T cells, reduced helper T-cell activity and response to antigens, reduced numbers of B cells and B-cell response to antigens, reduced antibody response, reduced phagocytosis, and reduced Toll-like receptors on phagocytic cells. (8) Feeding tubes, these reduce salivary flow and subsequently alter oropharyngeal colonization in tube-fed patients, but gastroesophagal reflux disease has also been shown to be increased in tube-fed patients and to predispose them to pneumonia. Increased incidence of oropharyngeal colonization with respiratory pathogens is also caused by impairment in salivary clearance [25]. The microbial etiology of AP involves *Staphylococcus aureus*, *Haemophilus influenza,* and *Streptococcus pneumoniae* for community-acquired AP and Gram-negative aerobic bacilli in nosocomial pneumonia [18]. It is worth bearing in mind the relative unimportance of anaerobic bacteria in AP [18]. Surprisingly, in the clinical setting, oropharyngeal dysphagia and aspiration are usually not considered etiologic factors in older patients with pneumonia [17, 18].

## 5. Treatment

Treatment of dysphagia in older patients varies greatly among centers. This variability can contribute to some controversy on the effect of swallowing therapy in preventing malnutrition and AP. In addition, there are a limited number of studies addressing these—unresolved—questions. A recent review found that there is insufficient data to determine the effectiveness of treatments for dysphagia in preventing AP in older adults [38]. In contrast, other authors found treatment of dysphagia is cost-effective and the use of dysphagia programs is correlated with a reduction in AP rates [27]. Management strategies for oropharyngeal dysphagia in older patients may be grouped into six major categories and simultaneously applied to the treatment of each patient [41]. During videofluoroscopy, a combination of strategies may be selected to compensate each patient's specific deficiency, and the usefulness of VFS in treating the patient's symptoms thus explored. Swallow therapy aims at improving the speed, strength, and range of movement of muscles involved in the swallow response and at modifying the mechanics of swallow to improve bolus transfer and avoid or minimize aspiration. It should be remarked that the largest body of literature cancers swallow therapy in older patients after strokes [27]. Furthermore, a recent systematic review on the effects of therapy in oropharyngeal dysphagia by speech and language therapists indicated that many questions remain about the actual therapeutic effects, even though some positive significant outcome studies have been published [42]. Many of these studies show diverse methodological problems, and because of the diversity in subject characteristics, therapies, and assessment instruments, the conclusions of most studies cannot be generalized or compared. We believe that management of dysphagia is not an exact science and a combination of clinical expertise and the best available evidence-based medicine is usually needed to manage elderly patients with oropharyngeal dysphagia [1, 27]. Preserved cognitive function is needed to apply some of the strategies. Nutritional and respiratory status should always be monitored in dysphagic patients in order to assess the efficacy of treatments.

### 5.1. Postural Strategies, Body and Head Positions

Verticality and symmetry should be sought during the patient's ingestion. Attention must be paid to controlling breathing and muscle tone. Postural strategies are easy to adopt—they cause no fatigue—and allow modification of oropharyngeal and bolus path dimensions. Anterior neck flexion (chin tuck) protects the airway [43–45]; posterior flexion (head extension or chin raise) facilitates gravitational pharyngeal drainage and improves oral transit velocity; head rotation (head turn maneuver) toward the paralyzed pharyngeal side directs food to the healthy side, increases pharyngeal transit efficacy, and facilitates UES aperture [44, 46], whereas head tilt to the stronger side prior to the swallow directs the bolus down to the stronger side by utilizing the effects of gravity; and deglutition in the lateral or supine decubitus protects against aspirating hypopharyngeal residues.

### 5.2. Change in Bolus Volume and Viscosity

In patients with neurogenic dysphagia and also in older patients, reductions in bolus volume and enhancement of bolus viscosity significantly improve safety signs, particularly regarding penetration and aspiration [10]. Viscosity is a physical property that can be measured and expressed in international system units by the name of Pa.s. The prevalence of penetrations and aspirations is maximal with water and thin fluids (20 mPa.s) and decreases with nectar (270 mPa.s) and pudding (3900 mPa.s) viscosity boluses [10]. Systematic videofluoroscopic studies found that increasing viscosity of liquids to pudding viscosity exerted such a dramatic reduction in the prevalence of penetrations and aspirations that routine introduction of dietary modifications in patients considered at risk of AP is logical [10, 27]. In addition, clinical studies also found dietary modifications can reduce the risk of AP [27]. Patients with decreased efficiency of deglutition need dietary adjustments to concentrate their caloric and protein requirements in the low volume of food they can swallow. Modifying the texture of liquids is particularly important to ensure that patients with neurogenic or ageing-associated dysphagia remain adequately hydrated and aspiration-free [2]. This may be easily achieved by using appropriate thickening agents [10].

### 5.3. Neuromuscular Praxis

The goal is to improve the physiology of deglutition (the tonicity, sensitivity, and motility of oral structures, particularly the lips and tongue, and pharyngeal structures). Lingual control and propulsion may be improved by using rehabilitation and biofeedback techniques [16]. Improved isometric strength after two months of progressive resistance lingual exercises has proved to correspond with spontaneous increased pressure generation during swallowing in stroke patients, thus showing significant improvement in swallowing function and dietary intake [16]. Of late, the rehabilitation of hyoid muscles with cervical flexion exercises (Shaker exercise) has been shown to improve hyoid and laryngeal elevation, to increase UES aperture, to reduce pharyngeal residue, and to improve dysphagia symptoms in patients with neurogenic dysphagia [47]. The management of patients with impaired UES aperture as a consequence of propulsive deficiencies should be basically oriented to increase bolus propulsion force and to rehabilitate the extrinsic mechanisms of UES aperture, particularly the activity of hyoid muscles [47]. The tongue-holding or Masako manoeuvre is presumed to compensate for the reduction in tongue base-pharyngeal wall contact in swallowing, thus contributing to an increased anterior movement of the posterior pharyngeal wall during swallowing. However, the use of the manoeuvre per se, which inhibits posterior retraction of the base of tongue, results in increasing the pharyngeal residue after the swallow. Another motor treatment for improving muscles strength is neuromuscular electrostimulation (NMES). The first study using NMES in dysphagic patients was performed by Freed et al. [48]. Since then, several studies have been published with controversial therapy outcome [49–52], probably due to the diversity in treatment parameters (frequency, pulse duration, or treatment intensity) and lack of a standard protocol for the use of NMES. However, although NMES as an adjunct to standard treatment is still controversial, a meta-analysis showed a small but significant treatment effect for transcutaneous NMES on patients with dysphagia [53].

### 5.4. Specific Swallowing Manoeuvres

These are manoeuvres the patient must be able to learn and perform in an automated way. Each manoeuvre is specifically directed to compensate specific biomechanical alterations [1, 41].

**Figure 6:**
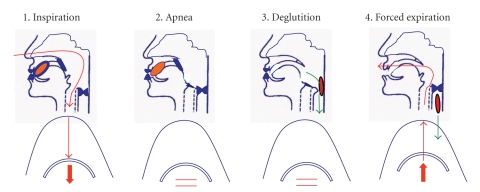
Diagrams showing the four steps of supraglottic swallow to protect the airway from aspiration. Commands for the patient are: (1) Take a deep breath, (2) Hold your breath, (3) Hold your breath while swallowing, (4) Cough immediately after you swallow.

**Figure 7:**
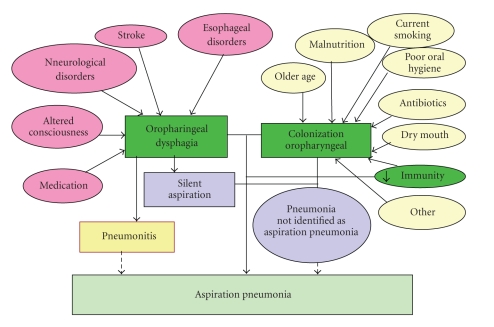
Risk factors for oropharyngeal colonization by respiratory pathogens and aspiration pneumonia in older people.

**Supraglottic and Super Supraglottic Swallow**

its aim is to close the vocal folds before and during deglutition in order to protect the airway from aspiration, and by coughing immediately after the swallow to clear any residue. The difference between these related manoeuvres is the degree of effort in the preswallow breath-hold. The super supraglottic swallow requires an effortful breath-hold, whereas the supraglottic swallow requires a breath-hold with no extra effort. It is useful in patients with penetrations or aspirations during the pharyngeal stage or slow pharyngeal motor pattern (Figure 6).

**Effortful, Forceful, or Hard Swallow**

Its aim is to increase the posterior motion of the tongue base during deglutition in order to improve bolus propulsion. It is useful in patients with low bolus propulsion [1, 41].

**Double Deglutition**

Its aim is to minimize postswallow residue before a new inspiration. It is useful in patients with postswallow residue [41].

**Mendelsohn Manoeuvre**

It allows for increased extent and duration of laryngeal elevation and therefore increased duration and amplitude of UES aperture [41].

### 5.5. Surgical/Drug-Based Management of UES Disorders

Identifying an obstructive pattern at the UES allows patient management using a surgical cricopharyngeal section [54] or an injection of botulin toxin [55]. Impaired neural UES relaxation observed in spastic neurological diseases such as Parkinson disease or brain injury is characterized by delayed or absent swallow response, short hyoid motion, weak bolus propulsion, and reduced or even absent neuromuscular relaxation and reduced sphincter compliance on manometry [56]. Treatment must combine treatment of neurogenic dysphagia and improvement of neuromuscular relaxation of the sphincter. Efficacy of cricopharyngeal myotomy in patients with impaired swallow response is fair to poor and injection of botox in the sphincter could be a therapeutic alternative for these patients. Patients with impaired UES opening associated with Zenker's diverticulum or isolated cricopharyngeal bars show normal swallow response, wide hyoid motion, and strong bolus propulsion and reduced sphincter compliance caused by sphincter fibrosis [57]. Treatment of this group of patients is surgical and combines cricopharyngeal myotomy and resection of the diverticulum. Surgical results in older patients with Zenker's diverticulum and preserved swallow response are excellent [57].

### 5.6. Sensorial Enhancement Strategies

Oral sensorial enhancement strategies are particularly useful in patients with apraxia or impaired oral sensitivity (very common in older patients) [41]. The aim of these strategies is the initiation or acceleration of the oropharyngeal swallow response. Most sensorial enhancement strategies include a mechanical stimulation of the tongue, bolus modifications (volume, temperature, and taste), or a mechanical stimulation of the pharyngeal pillars. Acid flavors such as lemon or lime [58, 59], and cold substances such as ice cream or ice [60], trigger the mechanism of deglutition, but may not reach clinical or statistical significance even after intense training.

### 5.7. Pharmacology of Swallow Response in Older People

Several drugs, most centrally acting, can elicit oropharyngeal dysphagia in older people. Neural activity in the nucleus tractus solitarius (NTS) is inhibited by *γ*-aminobutyric acid (GABA) [61, 62], and benzodiazepine administration can potentiate GABA system at CNS and cause dysphagia [63]. Ethanol also acts in the CNS binding to the GABA_A_ receptor and alcohol ingestion can predispose to oropharyngeal aspiration [64]. Neuroleptics are widely used in the older demented population for control of aggressive or disruptive behaviour, and doapmine antagonists like phenotiazines and haloperidol can impair swallow function. Moreover, extrapyramidal signs and xerostomia are common adverse effects of these drugs clearly associated with dysphagia [65, 66]. Use of neuroleptics is also associated with a 60% greater risk of pneumonia [67]. 

Studies using pharmacological stimulant agents also show some promising positive effects [38]. Several types of pharmacological and mechanical stimulation increase the concentration of Substance P (SP) in saliva and improve the swallowing reflex and cough-reflex sensitivity. The increase in serum SP with volatile black pepper oil or capsaicin might be closely related to improvement of the swallow response [68–70]. Capsaicin and piperine (active substance from black pepper) act as transient receptor potential channel vanilloid 1 (TRPV1) agonists. TRPV1 is widely expressed on sensory neurons innervating pharynx and larynx, projecting to NTS and colocalizes with SP [71]. Other stimulants of TRPV1, like heat and acid, have also been reported to improve swallowing [58, 59, 72]. Moreover, intervention with an angiotensin converting enzyme inhibitor also resulted in an increase in serum SP and reduced the incidence of AP [73, 74]. Use of a dopamine agonist such as amantadine and a folic acid supplement known to activate dopaminergic neurons also prevented AP [75]. Higher doses of _L_-dopa may reduce swallowing abnormalities [76]. The development of physical or drug-based strategies to accelerate the swallow response is a relevant field of research for the management of neurogenic dysphagia and ageing-associated dysphagia [1].

### 5.8. Percutaneous Endoscopic Gastrostomy

Videofluoroscopy will help in *treatment selection *depending upon the severity of efficacy or safety impairment in each patient: (a) patients with mild efficacy alterations and correct safety may have a family-supervised restriction-free diet; (b) in patients with moderate alterations, dietary changes will be introduced aiming at decreasing the volume and increasing the viscosity of the alimentary bolus; (c) patients with severe alterations will require additional strategies based upon increased viscosity and the introduction of postural techniques, active manoeuvres, and oral sensorial enhancement; and (d) there is a group of patients with alterations so severe that they cannot be treated despite using rehabilitation techniques; in these patients, VFS objectively demonstrates the inability of the oral route and the need to perform a percutaneous endoscopic gastrostomy (PEG) [2]. However, there is little evidence that nonoral feeding reduces the risk of aspiration [27]. Even though no absolute criteria exist, a number of dysphagia teams have indicated gastrostomy in: (a) patients with severe alterations of efficacy during the oral or pharyngeal stages, or with malnutrition; (b) patients with safety alterations during the pharyngeal stage that do not respond to rehabilitation; and (c) patients with significant silent aspirations, particularly in neurodegenerative conditions. For long-term nutritional support, PEG should be preferred to nasogastric tubes since it is associated with less treatment failure, better nutritional status and may also be more convenient for the patient [40]. In patients with severe neurological dysphagia, tube feeding has to be initiated as early as possible [16]. For most patients requiring gastrostomy a small percentage of food may still be safely administered through the oral route [2].
